# (Micro)saccade-related potentials during face recognition: A study combining EEG, eye-tracking, and deconvolution modeling

**DOI:** 10.3758/s13414-024-02846-1

**Published:** 2024-01-31

**Authors:** Lisa Spiering, Olaf Dimigen

**Affiliations:** 1 Department of Psychology, Humboldt-Universität zu Berlin, Berlin, Germany; 2 Department of Experimental Psychology, University of Oxford, Oxford, UK; 3 Present Address: Department of Psychology, University of Groningen, Grote Kruisstraat 2/1, 9712 TS Groningen, The Netherlands

**Keywords:** Early posterior negativity (EPN), Face processing, Refixations, Microsaccades, Eye tracking, Emotional facial expressions, Linear deconvolution modeling

## Abstract

Under natural viewing conditions, complex stimuli such as human faces are typically looked at several times in succession, implying that their recognition may unfold across multiple eye fixations. Although electrophysiological (EEG) experiments on face recognition typically prohibit eye movements, participants still execute frequent (micro)saccades on the face, each of which generates its own visuocortical response. This finding raises the question of whether the fixation-related potentials (FRPs) evoked by these tiny gaze shifts also contain psychologically valuable information about face processing. Here, we investigated this question by corecording EEG and eye movements in an experiment with emotional faces (happy, angry, neutral). Deconvolution modeling was used to separate the stimulus ERPs to face onset from the FRPs generated by subsequent microsaccades-induced refixations on the face. As expected, stimulus ERPs exhibited typical emotion effects, with a larger early posterior negativity (EPN) for happy/angry compared with neutral faces. Eye tracking confirmed that participants made small saccades in 98% of the trials, which were often aimed at the left eye of the stimulus face. However, while each saccade produced a strong response over visual areas, this response was unaffected by the face’s emotional expression, both for the first and for subsequent (micro)saccades. This finding suggests that the face’s affective content is rapidly evaluated after stimulus onset, leading to only a short-lived sensory enhancement by arousing stimuli that does not repeat itself during immediate refixations. Methodologically, our work demonstrates how eye tracking and deconvolution modeling can be used to extract several brain responses from each EEG trial, providing insights into neural processing at different latencies after stimulus onset.

Under natural conditions, humans actively seek out relevant visual information with several eye movements per second. For example, while looking at a face, we might direct our gaze towards the most informative facial features to evaluate another person’s current emotional state. More generally, it seems that complex visual objects such as faces are not always fully processed during a single glance, but rather across multiple subsequent fixations (Hsiao & Cottrell, 2008).

In contrast to natural viewing conditions, electrophysiological (EEG) experiments on face recognition typically require participants to maintain a prolonged fixation. However, abundant evidence suggests that even under the strictest of fixation instructions, oculomotor exploration behavior continues at a miniature scale during EEG experiments in the form of small and often involuntary (micro)saccades (Dimigen et al., 2009; Yuval-Greenberg et al., 2008). These saccades typically remain unnoticed by the experimenter since the small rotation of the eyeballs produces corneoretinal artifacts that remain below common detection thresholds in the electrooculogram (EOG). In addition to these artifacts, however, each of the small gaze shifts can also generate considerable visuocortical activity in the EEG (Dimigen et al., 2009; Gaarder et al., 1964), at least if the stimulus is of sufficient size and contrast (Armington et al., 1967; Gaarder et al., 1964).

In the current work, we used EEG, eye-tracking, and a linear deconvolution technique to test whether it is possible to fully separate these microsaccadic brain potentials from the temporally overlapping potentials elicited by the earlier stimulus onset. If possible, this would help EEG researchers to control for potential confounds from microsaccades in their data. More interestingly, however, in a second step, we also tested whether the fixation-related brain potentials (FRPs) generated by each of these small gaze shifts can be used to “probe” the ongoing state of neural stimulus processing during the trial.^1^ To explore this possibility, we tested whether the microsaccadic FRPs measured during a standard face recognition experiment are still sensitive to the facial expressions shown by the presented face, in the same way as traditional stimulus-locked ERPs (Schindler & Bublatzky, 2020). If this were to be the case, overlap-corrected microsaccade-induced brain activity could serve as a new type of neural marker for attentional, affective, or cognitive processes.

## “Pinging” neural states with (micro)saccadic brain activity?

As noted above, there is now much evidence that during common EEG paradigms, visual areas are frequently reactivated by microsaccades (Dimigen et al., 2009; see also Tse et al., 2010) or small exploratory saccades (Dimigen & Ehinger, 2021) on the stimulus.^2^ The most prominent component of the resulting FRP waveform is the lambda response, a positive potential that peaks ~90 ms after the end of the gaze shift. The lambda response shares many features with the P1 component in visually evoked potentials (VEPs; Kazai & Yagi, 2003; Thickbroom et al., 1991), especially in VEPs induced by pattern movement (Thickbroom et al., 1991). It is clear that the lambda response is primarily visual in nature, as also evident by its sensitivity to low-level stimulus features (such as luminance contrast; Gaarder et al., 1964) and its absence or attenuation in darkness (Billings, 1989; Fourment et al., 1976). Nevertheless, the fact that microsaccade-related brain potentials are present in the vast majority of trials in common EEG paradigms (Dimigen et al., 2009; Meyberg et al., 2015; Yuval-Greenberg et al., 2008) raises the questions of whether these potentials can be treated not just as artifacts (Yuval-Greenberg et al., 2008), but as a potential source of information.

One possibility is that the brain potentials produced by each of these small refixations are confined to early stages of visuocortical processing and that they lack cognitively-modulated or “endogenous” ERP components. This may also be due to rapid adaptation to the refixated stimulus (e.g., categorical adaptation of FRPs; Auerbach-Asch et al., 2020; Gert et al., 2022). An alternative possibility, however, is that FRPs from small saccades on the stimulus still reflect ongoing processes of attention and cognition. In the latter case, these potentials—if statistically separable from the stimulus ERP—might actually be useful to “probe” the neural system at different latencies after stimulus onset.

Task-irrelevant but contrast-rich probe stimuli have been successfully used in EEG research to assess the locus of covert attention (e.g., Hillyard & Anllo-Vento, 1998; Luck et al., 1993) and to decode otherwise “silent” working memory representations (Wolff et al., 2015, 2017). Since microsaccades generate visual transients that can be as strong as those from passive visual stimulation (Dimigen et al., 2009) they might be similarly useful for probing or “pinging” (Wolff et al., 2017) ongoing neural states. Evidence in favor of this idea comes from Meyberg et al. (2015), who analyzed microsaccades during the cue–target interval of a standard attentional cueing task. They found that not just the VEPs to externally flashed probes but also the brain waves generated by microsaccades reflected the cued locus of covert attention. Using the latter, it was also possible to dissociate covert attention, as reflected in the hemispheric lateralization of microsaccadic potentials, from overt attention, as reflected in the direction of microsaccades (for a similar finding, see Liu et al., 2022).

These results indicate that it may be feasible to use the omnipresent microsaccades to obtain neural markers beyond those contained in the stimulus-locked EEG. However, whereas the microsaccades in the attention experiments described above typically occurred a few seconds after the last stimulus presentation, we first have to deal with the problem of overlapping potentials.

## Deconvolution modeling can separate overlapping responses

A major challenge for analyzing co-registered EEG/eye-tracking data is that the neural responses to stimulus onset overlap with those to subsequent fixations on the stimulus. Without correction, the stimulus-ERP waveforms will therefore be distorted by FPRs and vice versa (e.g., Coco et al., 2020; Devillez et al., 2015; Gert et al., 2022; Meyberg et al., 2017). In addition to this overlap problem in the time domain, microsaccades also affect stimulus-related EEG analyses in the frequency domain, for example, by resetting (Dimigen et al., 2009; Dimigen, et al., 2011b; Gao et al., 2018) and lateralizing (Liu et al., 2023) ongoing alpha oscillations.

A promising approach to address these overlap problems is linear deconvolution modeling, also known as finite impulse response deconvolution (Dale & Buckner, 1997). In this framework, within a large regression model, each observed EEG sample is understood as the summation of overlapping responses by different experimental events (Dandekar et al., 2012; Devillez et al., 2015; Dimigen & Ehinger, 2021; Kristensen et al., 2017; N. J. Smith & Kutas, 2015b). Because the temporal distance between subsequent experimental events (e.g., stimulus onsets and microsaccades) varies naturally from trial to trial, it is possible to statistically separate the potentials related to each type of event. Additionally, the model can account for both linear and nonlinear (Ehinger & Dimigen, 2019) influences of continuous event properties on the EEG; for example, it can account for the nonlinear effect of saccade size on the FRP waveform (Yagi, 1979). After solving the model, the resulting regression coefficients can be analyzed just like conventionally averaged ERPs (N. J. Smith & Kutas, 2015a).

In summary, the linear deconvolution framework is promising to separate activity from multiple events within the same trial. In the current study, we build on previous work (e.g., Dimigen & Ehinger, 2021; Kristensen et al., 2017) to test whether we can fully separate stimulus- and microsaccade-related brain signals and whether the latter can then be used as a new type of probe for attentional and affective processing.

## Emotional facial expressions modulate stimulus-locked ERPs

In the current work, we explored these questions by focusing on the processing of emotional facial expressions. Effects of a face’s emotional valence (e.g., angry vs. neutral expression) are well-established in the literature where at least two prominent ERP components have been linked to the early and late processing of facial emotions (Schacht & Sommer, 2009; Schindler & Bublatzky, 2020):

The first and early component is the early posterior negativity (EPN), a negative deflection largest over bilateral occipitotemporal electrodes that differentiates emotionally neutral stimuli from those with a positive or negative valence (Schacht & Sommer, 2009; Schupp et al., 2004, 2006). The EPN typically begins around 150 ms after stimulus onset and reaches a maximum between 200–300 ms (Schupp et al., 2006), although it can last up to 600 ms poststimulus (Rellecke et al., 2012). The EPN is larger for emotionally arousing stimuli (such as faces with an angry or happy expression) and this is commonly assumed to reflect a reflex-like allocation of attention towards emotionally arousing stimuli leading to their enhanced sensory encoding. This may reflect an innate predisposition for emotional faces to capture processing resources (Junghöfer et al., 2001; Schacht & Sommer, 2009). Consequently, the EPN appears automatically regardless of the task or depth of stimulus processing (Rellecke et al., 2011, 2012). Since the EPN has consistently been shown to be modulated by facial expressions (Schindler & Bublatzky, 2020), we considered this component to be a suitable proxy to address our more general question of whether attentional or affective modulations are still present in the neural response elicited by microsaccades on the face.^3^

A second component linked to facial emotion processing is the late positive potential (LPP), a centroparietal positivity that emerges at around 350-500 ms post-stimulus (Schacht & Sommer, 2009; Schupp et al., 2006) and is larger for emotional stimuli. LPP effects have been observed in FRPs collected during the free viewing of emotional scenes, at least if the task requires an explicit arousal or valence rating (Simola et al., 2013, 2015). Unlike the EPN, the LPP is believed to reflect higher-level, elaborative, and nonautomatic stages of the encoding of emotional stimuli. As such, it is often absent if the task is superficial or if emotion is task-irrelevant, as it was the case in the current study. We therefore did not anticipate LPP effects in the present data.

## The present work

To summarize, the first aim of our study was to test whether we can use (non)linear deconvolution modeling to cleanly separate stimulus-locked responses from overlapping responses by small saccades. These genuine but often “hidden” cortical responses pose inferential hazards, since they are not removed by ocular correction algorithms (like independent component analysis [ICA]). If saccade rates or directions differ between conditions, these potentials can also distort effects in stimulus ERPs (Dimigen et al., 2009). Separating stimulus- from saccade-related brain activity should also lead to an improved signal-to-noise ratio.

Our second aim was to investigate whether after overlap-correction, the microsaccades themselves could be exploited as a source of information. More specifically, we examined whether the FRPs elicited by facial refixations merely reflect low-level changes in retinal stimulation or also a (re)processing of the face’s affective contents. In the latter case, we might be able to (1) extract multiple useful neural responses from each trial and (2) track the time course of affective processing via microsaccades at different latencies.

To address both questions, we reanalyzed data from a previously published experiment in which emotional expressions were displayed by static and dynamic faces (Bagherzadeh-Azbari et al., 2022). We chose this experiment because it included simultaneous eye-tracking/EEG recordings and because faces were presented for 2,000 ms in half of the trials. These relatively long trials allowed us to compare the neural responses following the first microsaccade on the face to those following microsaccades later in the trial. We hypothesized that the first microsaccade on the face—which typically happens 200–250 ms after stimulus onset (Engbert & Kliegl, 2003)—would still be crucial for ongoing face processing (Hsiao & Cottrell, 2008) and might therefore show the same arousal-related sensory enhancements as stimulus-locked ERPs (i.e., a larger EPN to happy and angry faces). In contrast, we expected EPN effects to be weak or absent for saccades late in the trial.

## Methods

### Participants

We analyzed the data of a face classification experiment previously reported in Bagherzadeh-Azbari et al. (2022). In the study, twenty university students (12 female; age range: 18 to 44 years, *M* = 24.40 years, *SD* = 6.03) participated in the experiment for course credit or monetary remuneration. According to the Edinburgh Handedness Inventory (German version; Oldfield, 1971), all but one participant were right-handed (mean handedness score = +91.40, *SD* = 24.57) and all participants self-reported normal or corrected-to-normal visual acuity. Before participating in the experiment, participants provided written informed consent as approved by the departmental ethics review board of the Department of Psychology at Humboldt-University.

### Stimuli

**Fig 1:**
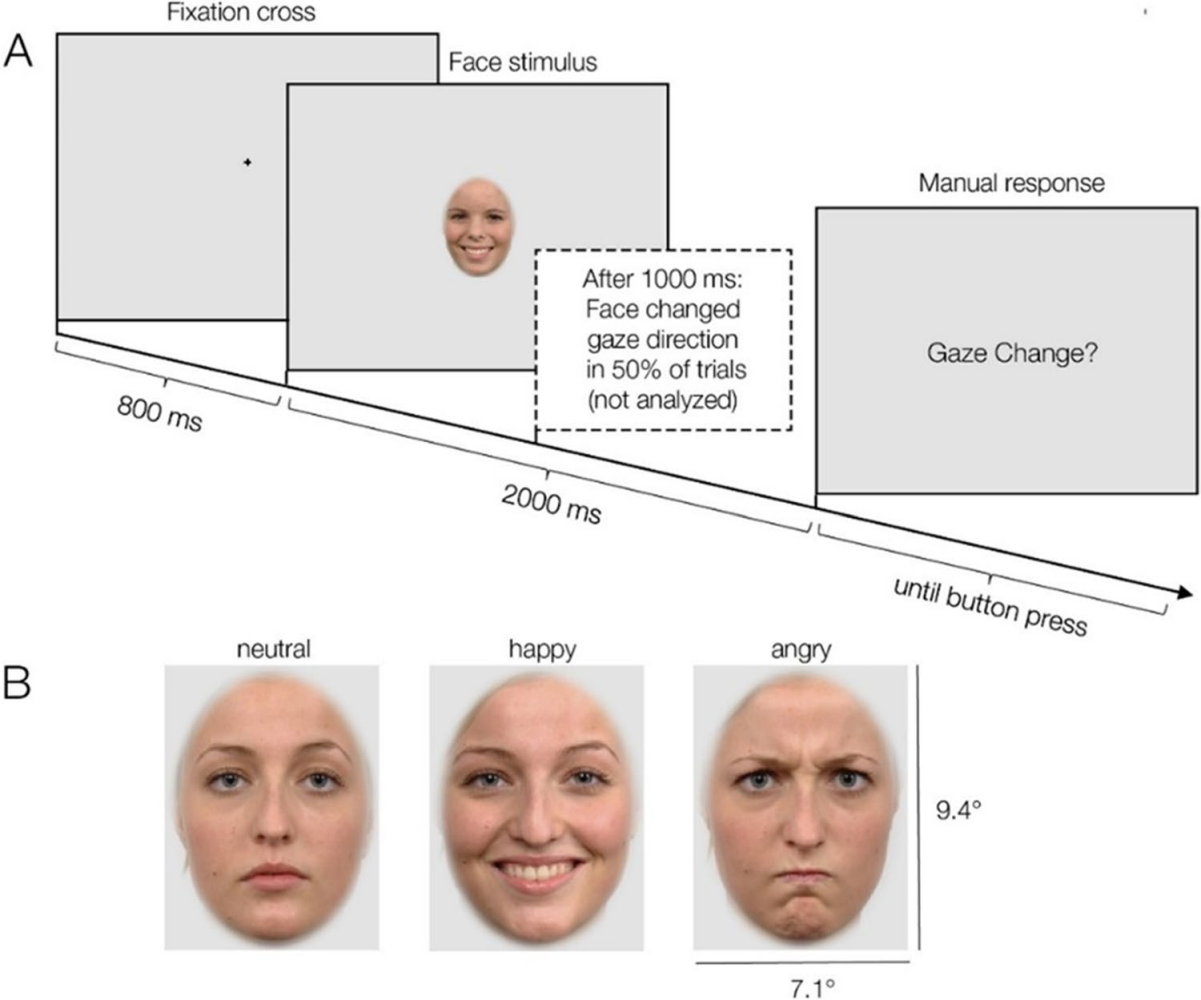
Trial scheme and example stimuli. **A** Participants were instructed to fixate a central cross shown for 800 ms. Afterwards, a face with a neutral, happy, or angry facial expression was presented for 2,000 ms. The participant’s task was to indicate whether or not the gaze direction of the face (direct forward gaze, leftward, or rightward averted) changed during the trial, which happened in half of the trials after 1,000 ms (these trials were not analyzed). **B **Shown here are the three emotional expressions for an example face with a direct (forward) gaze.

Images of faces of 36 individuals (18 female, 18 male), were selected from the Radboud Faces Database (Langner et al., 2010). Each stimulus showed a frontal-view color image of a Caucasian face. External facial features (e.g., neck and hairline) were removed by a standard oval aperture (see Fig. 1). During the experiment, each individual’s face was presented in nine different versions. It was shown with three different emotional expressions (neutral, angry, and happy) and also with three different gaze directions (the face looked directly at the observer, had an averted gaze position looking to the left, or an averted gaze position looking to the right). At the viewing distance of 60 cm, each face subtended 9.41° vertically and 7.07° horizontally. Both the size and the screen location of the presented faces were carefully standardized such that the eyes of the faces always appeared in the same screen position across trials. Figure 1B shows three example stimuli. More details on the creation of the face stimuli are provided in Bagherzadeh-Azbari et al. (2022).

### Procedure

Participants were seated in an acoustically and electrically shielded cabin in front of a 22-inch cathode ray tube monitor (Iiyama Vision Master Pro 510, vertical refresh rate: 160 Hz, resolution: 1,024 × 760 pixel). Following preparation of the EEG, participants first performed a 7-min calibration routine during which they made eye blinks as well as 15° eye movements in all four cardinal directions. These isolated saccades were later used by the ocular artifact correction procedure (see section Preprocessing). This calibration task was followed by the face experiment.

The trial scheme of the experiment is illustrated in Fig. 1A. Each trial started with the display of a black fixation cross (0.72° × 0.72°) on a homogeneous gray background. The fixation cross was presented 1.44° above the screen center and therefore centered on the nasion (bridge of the nose) of the future face stimulus; the initial viewing position on the face was therefore always in-between the eyes.

After 800 ms, the fixation cross was then replaced with the face stimulus. Half of the trials were static no-change trials, which were used for the current analysis. In these trials, the face was presented for 2,000 ms and remained unchanged. The other half of trials were dynamic gaze-change trials, which were not analyzed here. In these trials, the face remained on screen for 1,000 ms, but was then replaced for the remaining 1,000 ms by an almost identical version of the face that only differed in terms of its gaze direction. For example, the face might initially look at the observer during the first second of the trial (direct gaze) but then avert the gaze to look away from the observer (or vice versa). In these gaze-change trials, only the eye region of the stimulus changed whereas the rest of the face—including the emotional expression—remained the same. After two seconds, the face was always replaced by a response screen (see Fig. 1A), which prompted the participant to report whether the face had changed its gaze position or not.

As mentioned, for the purpose of the current study, we only analyzed the no-gaze-change trials in which the face remained static for 2,000 ms. Because of the long presentation duration of the faces in this condition, these trials provided an ideal opportunity to study the neural correlates of (micro)saccades executed at different latencies after stimulus onset.

The participant’s task was to watch the face and classify whether or not the face had changed its gaze direction during the trial. Participants were instructed to give their response only after the end of the trial using two manual response buttons operated with the left and right index fingers. The mean response accuracy was high (*M* = 97.8% correct, range across participants 92.8 to 100%, *SD* = 1.8%) and not further analyzed here. In case of incorrect or premature responses (before the face had disappeared), a red error message was shown. The participant’s button press initiated the next trial, which again started with the fixation cross.

Participants received written instruction to focus on response accuracy, to fixate on the central fixation cross while it was visible, and to avoid blinking their eyes while the face was shown. Instead, they were encouraged to blink after the end of the 2-second face presentations.

The experiment comprised 864 trials, divided into 8 blocks, plus an additional 12 practice trials before the experiment. Facial emotion (neutral, happy, angry) and trial type (no-change vs. gaze-change) were counterbalanced and these six conditions were shown equiprobably during the experiment in an individually randomized order. Within the change-trials (not analyzed), gaze changes leading to an averted gaze position (i.e., direct-to-averted gaze, left-averted to right-averted gaze, right-averted to left-averted gaze) and changes leading to a direct gaze position (i.e., left-to-direct, right-to-direct) occurred equally often. The same was true for averted gaze positions towards the left versus right. The experiment was implemented using Presentation® software (Version 18.10, Neurobehavioral Systems, Inc., Berkeley, CA).

### Eye-movement recording

Binocular eye movements were recorded at a 500 Hz rate with a video-based eye-tracker (iView X Hi-Speed 1250, Sensomotoric Instruments GmbH, Germany). Head movements were restricted by the chin and forehead rest of the eye-tracker’s tower mount. The system was calibrated and validated with a 9-point grid before every block or whenever necessary during the experiment. Validations were accepted if the mean vertical and the mean horizontal validation error were both <1°.

### Electrophysiological recordings

Electrooculogram (EOG) and EEG were recorded from 47 passive Ag/AgCl electrodes using BrainAmp amplifiers (Brain Products GmbH, Gilching, Germany). Forty-two electrodes were mounted on an elastic textile cap (EasyCap, Herrsching, Germany) at positions of the international 10/10 system; the exact montage is documented in Dimigen (2020, online supplement). External electrodes were placed on the left (M1) and right (M2) mastoid; four EOG electrodes were placed at the outer canthus and infraorbital ridge of each eye. An electrode at FCz served as ground. Impedances were kept below 10 kΩ. To avoid pressure artifacts from contact with the eye-tracker’s forehead rest, foam rings were fitted around the prefrontal (Fp1/2) electrodes. During recording, all signals were referenced against electrode M1. Electrophysiological signals were sampled at 500 Hz at a time constant of 10 s with an online low-pass filter set to 100 Hz.

### EEG preprocessing and ocular artifact correction

Preprocessing of the EEG data was performed using EEGLAB (Version 13.6.5b; Delorme & Makeig, 2004) and the EYE-EEG toolbox (Version 0.81; Dimigen et al., 2011a). In a first step, the EEG was digitally re-referenced to an average reference, thereby recovering the implicit reference (M1) as a recording electrode. Data was then bandpass-filtered between 0.1 to 45 Hz (−6 dB cutoffs) using EEGLAB’s windowed-sinc filter (*pop_eegfiltnew.m*) with default transition bandwidth settings. Ocular EEG artifacts were corrected using the Multiple Source Eye Correction method (MSEC; Berg & Scherg, 1994; Ille et al., 2002), as implemented in BESA (Version 6.0; BESA GmbH, Gräfeling, Germany). The MSEC method provides an excellent correction of corneoretinal and blink artifacts (Dimigen, 2020) but only partially removes the saccadic spike potential, the sharp biphasic spike of synchronized extraocular muscle activity peaking at saccade onset (Keren et al., 2010). Using the EYE-EEG toolbox, eye-tracking and EEG data were then synchronized based on shared trigger pulses sent frequently to both systems. The average synchronization error (misalignment of shared trigger pulses after synchronization) was <1 ms.

### Trial selection

For data analysis, we focused exclusively on no-change trials during which a completely static emotional face was continuously shown for 2,000 ms. This selection allowed us to study the FRPs elicited by saccades on the face at varying latencies without any confounds due to a change of the stimulus. Furthermore, in all analyses, we aggregated across the gaze direction displayed by the face (direct, averted-left, or averted-right), since this factor was of no relevance for the current research questions.

In a first step, we identified clean trials in which neither the eye-tracking data nor the EEG contained missing data or artifacts from −200 to 2,000 ms relative to stimulus onset. Three criteria were used to find clean trials: First, we rejected trials that included either an eye blink or gaze measurements outside of the stimulus image. Second, we excluded trials in which the mean gaze position during the 200 ms prestimulus interval was not within an invisible quadratic bounding box (side length: 3°) centered on the fixation cross (post hoc fixation check). Finally, we discarded trials that contained remaining non-ocular EEG artifacts (after ocular artifact correction), defined as any voltages exceeding ±120 μV relative to the baseline voltages at each channel. In the remaining clean trials (80% of all analyzed trials), saccade and fixation events were detected using the binocular version of the velocity-based microsaccade detection algorithm by Engbert and Kliegl (2003) as implemented in the EYE-EEG toolbox (velocity threshold: 5 median-based *SD*s, minimum saccade duration: 8 ms, binocular overlap required).

### Eye-movement analysis

We analyzed possible effects of facial emotion on five aspects of eye movement behavior: Saccade rate, saccade amplitude, and saccade direction (angle), as well as the vertical and horizontal fixation location within the face. Saccade rate was analyzed globally as a function of the emotion condition. For the four remaining dependent variables, measures were computed separately for the refixation following the first microsaccade on the face (called the “first fixation” in the following) and for all subsequent refixations on the face. For each type of saccade (first vs. subsequent) the four dependent measures were analyzed as a function of the emotional expression (neutral, happy, angry).

Since some of our eye movement measures have complex multimodal distributions (e.g., saccade angle is a circular predictor), we compared the distribution of each dependent variable across the three levels of *emotion* using pairwise nonparametric Kolmogorov–Smirnoff (KS) tests (i.e., angry vs. happy, happy vs. neutral, and angry vs. neutral). Per dependent variable, we corrected the resulting *p* values using the Bonferroni-correction (*p*_corr_) to account for the multiple pairwise comparisons. Saccade rate was corrected for 3 pairwise comparisons (across *Emotion* levels); all other eye movement measures were corrected for 6 pairwise comparisons (2 *Saccade Types* × 3 *Emotions*). We computed the mean and standard deviations of saccade angles using the *CircStat* toolbox for MATLAB (Berens, 2009).

### Linear deconvolution modeling

Stimulus- and fixation-related brain responses were modeled and statistically separated using linear deconvolution modeling with additional nonlinear spline predictors (for reviews see N. J. Smith & Kutas, 2015a, 2015b) as implemented in the *unfold* toolbox for MATLAB (Ehinger & Dimigen, 2019). In the following, we will only provide a brief and informal summary of this analysis approach. For details, the reader is referred to recent tutorial papers explaining this approach in detail (Dimigen & Ehinger, 2021; N. J. Smith & Kutas, 2015b). Technical details on the *unfold* toolbox are found in Ehinger and Dimigen (2019). Subsequently, we will document how we set up the specific model for the present analysis.

Compared with traditional ERP averaging, linear deconvolution modeling has two crucial advantages for analyzing experiments with eye movements (Auerbach-Asch et al., 2020; Dandekar et al., 2012; Devillez et al., 2015; Dimigen & Ehinger, 2021; Gert et al., 2022; Guérin-Dugué et al., 2018). First, the normal temporal variability between different oculomotor events (e.g., stimulus onsets and saccade onsets) can be used to statistically disentangle the overlapping brain responses produced by each type of event. Second, the model allows the researcher to statistically control for the effects of various nuisance variables that are known to influence eye movement-related brain responses (such as the saccade amplitude preceding a fixation). Because these waveforms are estimated within a regression framework rather than with classic averaging, they are sometimes referred to as regression ERPs (rERPs, N. J. Smith & Kutas, 2015a), or, in the case of fixation-related potentials, as regression FRPs. In the following, we will therefore refer to “rERPs” or “rFRPs” whenever we refer to waveforms obtained with the *unfold* toolbox.

One practical prerequisite of using linear deconvolution models is that the EEG recording is still continuous rather than cut into epochs. This is necessary so that the overlapping activity between all relevant experimental events can be considered in the estimation. To reduce computation time and the number of estimated parameters, the continuous artifact-corrected EEG was downsampled to 200 Hz. In this EEG dataset, we then included the stimulus onset events (coding the face onset event at the start of the trial) and the onsets of (re)fixations following microsaccades within the face. We only imported events from “clean” trials without missing data or residual artifacts (see above for screening criteria). To obtain the estimates for the non-overlapped rERPs and rFRPs, each channel of the continuous EEG signal was then modeled using a time-expanded design matrix in the *unfold* toolbox. Time-expansion means that we model the continuous EEG in a certain time window (here: −200 to 800 ms) around each experimental event (here: stimulus onset and fixation onsets). For each time point, for each type of event (stimulus or fixation), and for each predictor in our regression model (e.g., the emotion of the viewed face), we then add a column to the design matrix which codes the state of this predictor at this time point relative to the event. This time-expansion step, illustrated in Fig. 2 of Ehinger and Dimigen (2019), makes it possible to account for temporally overlapping effects of past and future events. This large regression model is then solved for the regression coefficients (or “betas”), which capture how much each event/predictor contributed to the measured EEG within the time expansion window. The resulting beta estimates can be plotted like an ERP waveform (N. J. Smith & Kutas, 2015a).

**Fig. 2:**
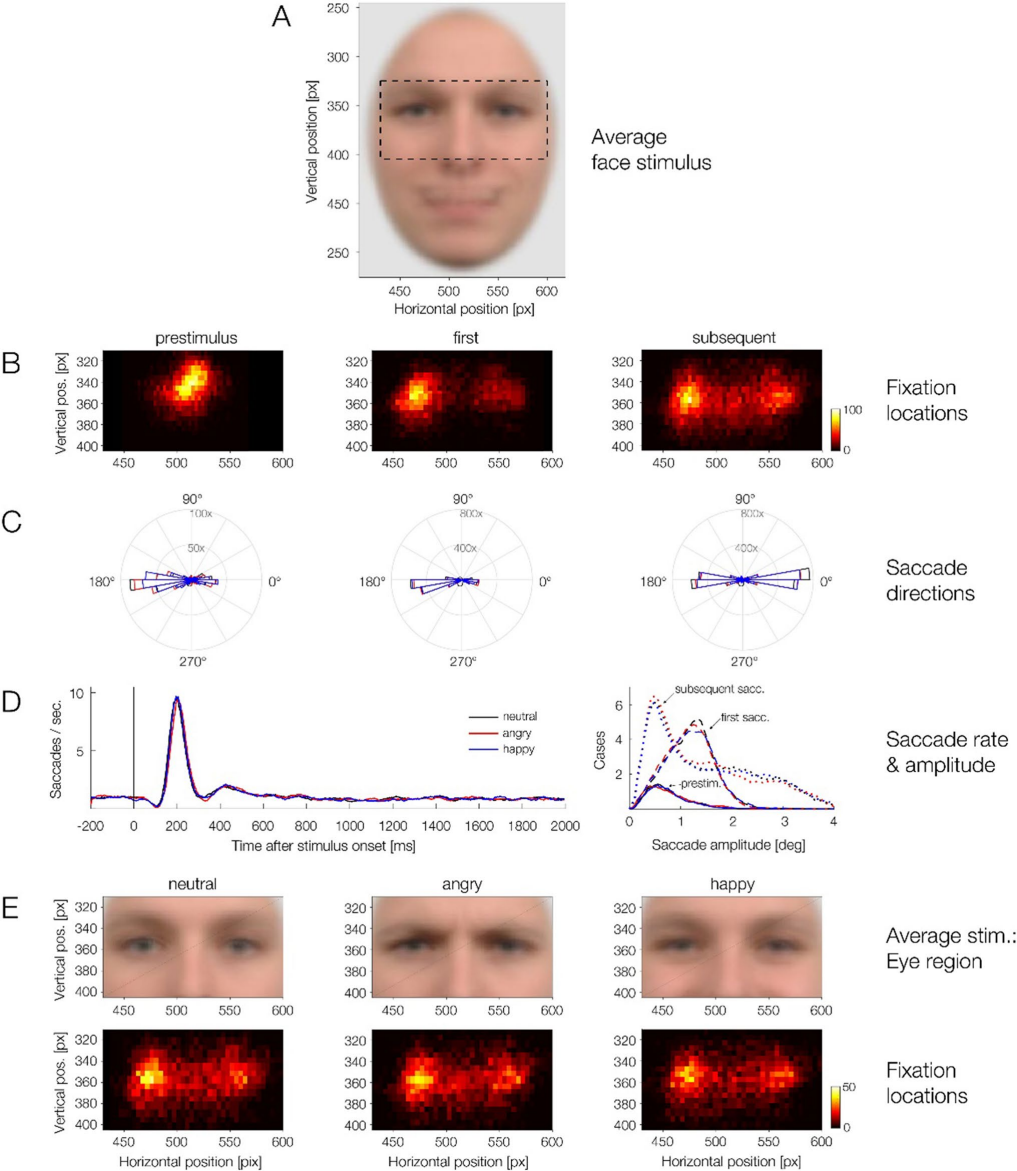
Properties of (micro)saccades detected during the trials. **A** Average of all presented face stimuli. The dashed rectangle around the eye region highlights the region for which fixation density heatmaps are shown in panels *B* and *E*. Most saccades occurred within this eye region. **B** Fixation density plots (“heatmaps”) of fixation locations during the prestimulus interval (−200 to 0 ms, left column), following the first saccade (middle) and following all subsequent saccades during the trial (right). **C** Polar shots show the distribution of saccade directions for the pre-stimulus saccades, the first saccades, and for subsequent saccades. **D** Rate of saccades over time (left) and distribution of saccade amplitudes (right). Results are shown separately for the three emotion conditions. **E** Fixation heatmaps, shown separately for the neutral, angry, and happy condition across the poststimulus interval (0–2,000 ms). (Color figure online)

Some predictors, such as saccade amplitude, have a strongly nonlinear influence on the neural response. Within the linear deconvolution framework, it is also possible to account for predictors that have nonlinear effects (N. J. Smith & Kutas, 2015b) by modeling their effects via a basis set of overlapping spline functions (cf. generalized additive modeling, GAM). For detailed tutorial reviews on how to model nonlinear effects within the deconvolution framework, the reader is referred to Dimigen and Ehinger (2021); Ehinger and Dimigen (2019); and N. J. Smith and Kutas (2015b).

In the following section, for clarity, we report the predictors used to model the brain responses elicited by stimulus onsets and fixation onset events as two separate subformulas. Please note, however, that these two formulas together specify one large regression model in which the regression coefficients (or “betas”) for all event types and predictors are estimated simultaneously. To model rERPs and rFRPs, we specified the following model, using a modified Wilkinson notation:

1. For stimulus onset events:

 rERP ~ 1 + cat(Emotion)

(2) For fixation onset events:

 rFRP ~ 1 + cat(Emotion) * cat(SaccadeType) + spl(SaccadeAmp,5) + FovealLum 

Here, the rERP waveform following stimulus onset events is simply modeled by an intercept term (1) and the categorical predictor *Emotion*, with the three levels of emotion categorically coded in the design matrix as 0 for neutral, 1 for angry, and 2 for happy. With the default treatment coding used by the *unfold* toolbox, the beta coefficients for the intercept term (1) simply correspond to the average rERP waveform elicited by the presentation of a neutral face. The resulting beta coefficients for the predictor *Emotion* then describe the additional effect produced by showing an angry or happy face, respectively. The betas of this predictor are therefore equivalent to a difference wave in a traditional ERP averaging analysis (e.g., with treatment coding, the betas for emotion level “2” are analogous to a difference wave “happy minus neutral”).

Within the same model, the brain responses following fixation onsets were modeled by a more complex formula: To model rFRPs, we again included an intercept term (1) as well as the categorical predictor *Emotion*, coded in the same way as for stimulus onsets. In addition, we added the categorical predictor *SaccadeType* that coded whether the current fixation followed either the first saccade (0) or any of the subsequent (1) saccades on the stimulus. In line with our hypothesis that emotion effects in FRPs might be stronger for the first fixation (i.e., for the fixation following the first saccade), we also allowed for an interaction between *Emotion* and *SaccadeType*.

Finally, two continuous covariates were included in the model to control for low-level nuisance effects on the rFRP: saccade amplitude (*SaccadeAmp*) and foveal image luminance (*FovealLum*). It is well-established that saccade amplitude has a strong influence on the shape of the postsaccadic brain response (e.g., Armington & Bloom, 1974; Yagi, 1979), with larger saccades followed by larger neural responses. Because this relationship is nonlinear (Boylan & Doig, 1989; Dandekar et al., 2012; Dimigen & Ehinger, 2021; Kaunitz et al., 2014; Ries et al., 2018) we included saccade amplitude as a nonlinear predictor modeled by a set of five spline functions (Dimigen & Ehinger, 2021).

Finally, as a second nuisance variable, we included the approximate luminance of the face stimulus at the currently inspected image location, which is also known to affect the post-saccadic neural response (Armington et al., 1967), because more luminant and more contrast-rich foveal stimuli generate larger lambda responses. For each fixation, foveal stimulus luminance was calculated within a circular patch with a radius of 2° centered on the current fixation location. Approximate foveal luminance was estimated by taking the mean of the channel-weighted RGB values (using MATLAB function *rgb2gray.m*) of all face image pixels within this region. Because the P1 amplitude is known to scale linearly with the logarithm of stimulus luminance (Halliday, 1982), this predictor was first log-transformed and mean-centered, and then added as a continuous predictor (*FovealLum*).

By solving the regression model for the betas (for each EEG channel), we obtained a time series of beta coefficients for each predictor in the design matrix. To obtain a waveform that corresponds to a traditional ERP curve (e.g., for angry faces), we can simply sum up the respective betas and also include the intercept term, which capture the overall waveshape of the ERP. For plotting rFRP waveforms, the two included nuisance predictors (saccade amplitude and foveal luminance) were evaluated at their respective mean values.

### Comparison to ERP averaging

Deconvolved potentials were compared with those obtained with traditional averaging. A useful feature of the *unfold* toolbox is that in addition to the overlap-corrected waveforms, it also provides the ERP/FRP averages that would result from the traditional averaging of the data, without controlling for overlapping potentials and covariates. To obtain these traditional averages, the continuous EEG was first cut into epochs of −200 to 800 ms around stimulus onsets and fixation onsets, respectively. In a second step, we generated the same design matrix of the model as for the deconvolution modeling, but without the time expansion step to control for overlapping potentials and without adding the continuous nuisance variables (saccade amplitude and foveal luminance) to the design matrix. This simple mass univariate regression model (N. J. Smith & Kutas, 2015a), which is equivalent to traditional averaging, was then again solved for the betas.

### Second-level EEG statistics

Both traditional averaging and deconvolution modeling provide waveforms at the single-subject level, which can then be tested for statistical significance at the group level. All of the second-level statistical analyses reported in the following were performed on the overlap-corrected (deconvolved) potentials.

#### Analyses of variance

As a first statistical approach, we computed repeated-measures ANOVAs in an a priori defined spatiotemporal region of interest (ROIs) using the *ez* package (Lawrence, 2016) for frequentist ANOVAs. As outlined in the Introduction, emotion effects are most reliably found on the EPN and LPP components, but LPP effects are often limited to tasks that require an explicit emotion decision (Rellecke et al., 2012). Since facial emotion was not task-relevant, we expected valence effects mainly on the EPN. To capture the EPN, following prior studies (e.g., Schupp et al., 2006), we used as the spatiotemporal ROI the average voltage at six occipitotemporal electrodes (P7/P8, PO7/PO8, PO9/PO10) in the time window from 200–300 ms.

The average voltage in this ROI was computed for the deconvolved rERP and rFRP waveforms and submitted to repeated-measures ANOVAs. In a first step, we ran a global repeated-measures ANOVA to test for emotion effects across two event types, that is, stimulus onset and fixation onsets. In this global ANOVA, we predicted average voltage in the prespecified ROI using the two within-subject factors *Emotion* (3 levels) and *Event Type* (2 levels: stimulus-locked/rERP, or refixation-locked/rFRP), as well as the interaction between these two main effects. For the purpose of this global ANOVA, we averaged the rFRPs across the factor *Saccade Type* (first vs. subsequent).

Since this global ANOVA produced a strong interaction between *Emotion* and *Event* type (see Results), we subsequently ran separate ANOVAs for each event type: For stimulus rERPs, the ANOVA only included the three-level factor *Emotion*. For the fixation-rFRPs, the ANOVA included the factors *Emotion* (3 levels), *Saccade Type* (2 levels: first or subsequent saccade) and the interaction term *Emotion* × *Saccade Type*. In case of significant effects, factor levels were compared with post hoc *t* tests.

#### Supplementary Bayesian analyses

As a supplementary analysis, we quantified the amount of evidence in favor of or against emotion effects in rERPs and rFRPs. For this, we conducted Bayesian ANOVAs using the BayesFactor package for Bayesian ANOVAs in R (Morey et al., 2015). An advantage of this Bayesian approach over a frequentist analysis is that it allows examining whether the data is more likely to have occurred under the null hypothesis (brain potentials do not differ between emotion conditions) or under the alternative hypothesis (brain potentials differ between emotion conditions).

The Bayesian ANOVA was calculated on the same EPN ROI window as the frequentist ANOVA. We first performed a factorial Bayesian ANOVA on stimulus rERPs on the within-subject factor *Emotion*. For rFRPs, we first aggregated across *Saccade Type* and then ran a factorial Bayesian ANOVA on the within-subject factor *Emotion.* The resulting Bayes factors (*BF*) were computed using the default Cauchy priors (*r* = 0.5 for the fixed effect of *Emotion*, and *r* = 1 for the random effect of subject). To interpret the resulting *BF*s, we used the classification by Lee and Wagenmakers (2013). The BayesFactor package estimates BFs with the Markov chain Monte Carlo (MCMC) algorithm (default number of samples = 10,000). Therefore, we report error percentages as an indication of the numerical robustness of the BF. Here, lower error percentages reflect a greater stability of the BF. Error percentages below 20% have been suggested as acceptable by van Doorn et al. (2021).

#### Cluster permutation tests

Since still relatively little is known about emotion effects in FRPs (Guérin-Dugué et al., 2018; Simola et al., 2013, 2015), it is possible that their spatiotemporal properties differ from those in traditional ERPs (for a comparison, see Simola et al., 2013). To test for emotion effects (Schindler & Bublatzky, 2020) also outside of the predefined spatiotemporal ROI of the EPN component, we ran an additional cluster permutation test. For this purpose, we used the threshold-free cluster-enhancement (TFCE) procedure, a data-driven permutation test that stringently controls for multiple comparisons across time points and channels. The TFCE procedure was originally developed to address the multiple-comparison problem with fMRI (S. M. Smith & Nichols, 2009) but subsequently adopted to M/EEG data by Mensen and Khatami (2013). Compared with earlier variants of cluster permutation tests (Maris & Oostenveld, 2007) the main advantage of TFCE is that the researcher does not need to set an arbitrary cluster-forming threshold. Instead, the cluster-enhancement process of TFCE can be thought of as adopting all possible clustering thresholds.

For the present purpose, we used the ANOVA variant of the TFCE algorithm as implemented in the *ept_TFCE* toolbox (https://github.com/Mensen/ept_TFCE-matlab). The test was conducted across all 46 channels and the entire latency range from 0 to 500 ms after stimulus/fixation onset, respectively. We again applied this analysis only on the deconvolved potentials. Factors were specified in the same way as for the traditional repeated-measures ANOVAs described further above: The TFCE-ANOVA for rERPs only included the factor *Emotion*, whereas the one for rFRPs additionally included *SaccadeType* and the *Emotion* × *SaccadeType* interaction. Whenever the TFCE-ANOVA revealed significant effects or interactions of the three-level factor *Emotion*, we also computed TFCE-based contrasts (*t* tests) between the respective emotion levels. Cluster-enhanced *F* or *t* values were compared against null distributions based on *n* = 5,000 random permutations of the condition labels.

## Results

### Eye movements

Figure 2 summarizes the eye movements during the task. Simultaneous eye tracking revealed that participants made small saccades in the vast majority (*M* = 97.9%) of trials. In trials with at least one saccade, the first saccade was usually (in 74.8% of cases) followed by at least one more subsequent saccade on the face. As shown in Fig. 2, the majority of saccades was aimed at the eye region of the stimulus face. Figure 2B and C present a more detailed visualization of the fixation locations at different latencies during the trial. As expected, during the pre-stimulus baseline interval (−200 to 0 ms), most saccades were still located near the fixation cross, although participants already showed some tendency for making anticipatory saccades towards the left (from the participants perspective) before stimulus onset. This is also visible in the polar histogram of saccade angles during the baseline period (see left polar plot in Fig. 2C; 64.87% of baseline saccades were directed leftward).

Once the face stimulus appeared, the first saccade was almost always directed horizontally, with a clear preference for leftward rather than rightward saccades (Butler et al., 2005; Hsiao & Cottrell, 2008; see Nuthmann & Matthias, 2014, for a similar bias in natural scenes). As the center panel of Fig. 2B shows, this initial saccade was typically aimed at the left eye of the stimulus face (69.76% of saccades directed leftwards). As the heatmaps in Fig. 2E show, this left-eye bias was present in all three emotion conditions. Subsequent saccades on the faces, following the initial saccade, were also predominantly horizontally oriented, but more widely distributed across the eye region, with a (slight) bias towards rightward saccades (52.69% directed rightwards, see right panel of Fig. 2C).

Saccades had a median amplitude of 1.23° (*SD* = 0.87), and this was quite similar for the first saccade (*M* = 1.21°, *SD* = 0.46) and subsequent saccades (*M* = 1.25°, *SD* = 1.02) on the face (Fig. 2D, right). The left panel of Fig. 2D shows the rate of saccades over time. Across the entire duration of the trial, the mean rate was 1.29 saccades per second. Following the onset of the stimulus, we observed the typical biphasic pattern (Engbert & Kliegl, 2003) consisting of an initial inhibition of saccades, followed by a strong rebound: Almost no saccades were observed at around 120 ms after face onset (peak of saccadic inhibition), but this was then followed by a strong increase in saccade rate, peaking shortly after 200 ms.

While the eye movements were generally highly similar for the three different facial emotions, a few eye movement parameters did show small but statistically significant differences. Table 1 shows the results of the KS tests on all eye movement measures, analyzed across the poststimulus interval from 0 to 2,000 ms. In terms of their vertical fixation location within the face, subsequent fixations on angry faces were distributed slightly differently as compared with those on neutral or happy faces (angry-neutral: *D* = 0.05, *p*_*corr*_ = 0.006; angry-happy: *D* = 0.04, *p*_*corr*_ = 0.03). However, these significant differences were numerical extremely small (on average <2 pixels or <0.07° in the mean values in both comparisons). Similarly, saccade amplitudes were slightly smaller in the angry as compared with both the neutral and happy conditions (first saccade: angry-neutral, *D* = 0.05, *p*_*corr*_ = 0.02; subsequent saccade: angry-neutral, *D* = 0.04, *p*_*corr*_ = 0.02; subsequent saccade: angry-happy: *D* = 0.05, *p*_*corr*_ = 0.001). Unstandardized effect size was again small; in terms of their median value, saccade amplitudes differed by less than 0.09° between emotion conditions. None of the other eye movement measures (saccade rate, saccade amplitude, saccade angle, horizontal or vertical fixation location) showed significant effects of *Emotion* (all *p*_*corr*_ > 0.05; see Table 1).

**Table 1:** Test statistics for effects of *emotion* on eye movements (KS-test results)

	Mean (*SD*)			Kolmogorov’s D			*p* value (corr.)		
Eye movement variable	Neutral	Angry	Happy	A–N	H–N	A–H	A–N	H–N	A–H
**Saccade rate** [per sec.]									
	1.28 (1.57)	1.27 (1.54)	1.26 (1.60)	0.10	0.07	0.08	.10	1.00	.52
**Saccade amplitude** [°]									
first	1.22 (0.45)	1.18 (0.45)	1.21 (0.47)	0.05	0.03	0.04	.02*	1.00	.30
subsequent	1.51 (1.01)	1.47 (1.01)	1.54 (1.03)	0.04	0.02	0.05	.02*	1.00	.001**
**Saccade angle** [°]									
first	158.70 (61.78)	156.04 (62.32)	155.89 (62.03)	0.03	0.03	0.02	1.00	1.00	1.00
subsequent	1.24 (78.36)	3.51 (77.85)	−0.80 (78.45)	0.02	0.03	0.02	1.00	.69	1.00
**Horizontal fixation location** [px]									
first	498.13 (35.72)	499.31 (35.65)	497.91 (35.91)	0.04	0.02	0.04	.58	1.00	.70
subsequent	512.47 (37.65)	512.99 (37.59)	512.21 (38.00)	0.02	0.01	0.02	1.00	1.00	1.00
**Vertical fixation location** [px]									
first	353.66 (15.33)	354.38 (14.99)	354.18 (15.98)	0.03	0.02	0.32	1.00	1.00	1.00
subsequent	359.63 (20.88)	361.62 (21.77)	361.39 (23.20)	0.05	0.02	0.04	.006**	1.00	.03*

N = neutral, A = angry, H = happy. Saccade angles were averaged using circular statistics (see Methods). A saccade angle of ±180° indicates a leftward saccade. Reported *p* values are Bonferroni corrected to adjust for the three (for saccade rate) or six (all other measures) pairwise comparisons per dependent variable; significant *p* values are highlighted in bold and marked with asterisks (*p < .05, **p < .01)

Taken together, the behavioral results show that in a traditional ERP experiment with a fixation instruction, participants made small saccades (of about 1.2° amplitude) in virtually every trial. Most properties of these eye movements were not modulated by the emotional content of the faces and the unstandardized effect sizes of the significant emotion effects were small. Instead, participants’ eye movements were overall rather stereotypical, with the first saccade (after about 200 ms) being typically aimed at one of the eyes of the face, a highly task-relevant part of the stimulus. This first saccade was often followed by one or more subsequent saccades, which often remained within the eye region of the face.

### Comparison of absolute brain responses with and without deconvolution

In most ERP studies, it is not considered that additional (micro)saccades happen during the trial. In a first step, we therefore assessed the impact of the overlapping FRPs from these saccades on the waveshapes of the stimulus-locked ERPs and vice versa. In particular, we compared the waveforms obtained with traditional averaging (ERPs/FRPs) with those obtained with deconvolution (rERPs/rFRPs).

#### Stimulus ERPs

**Fig. 3:**
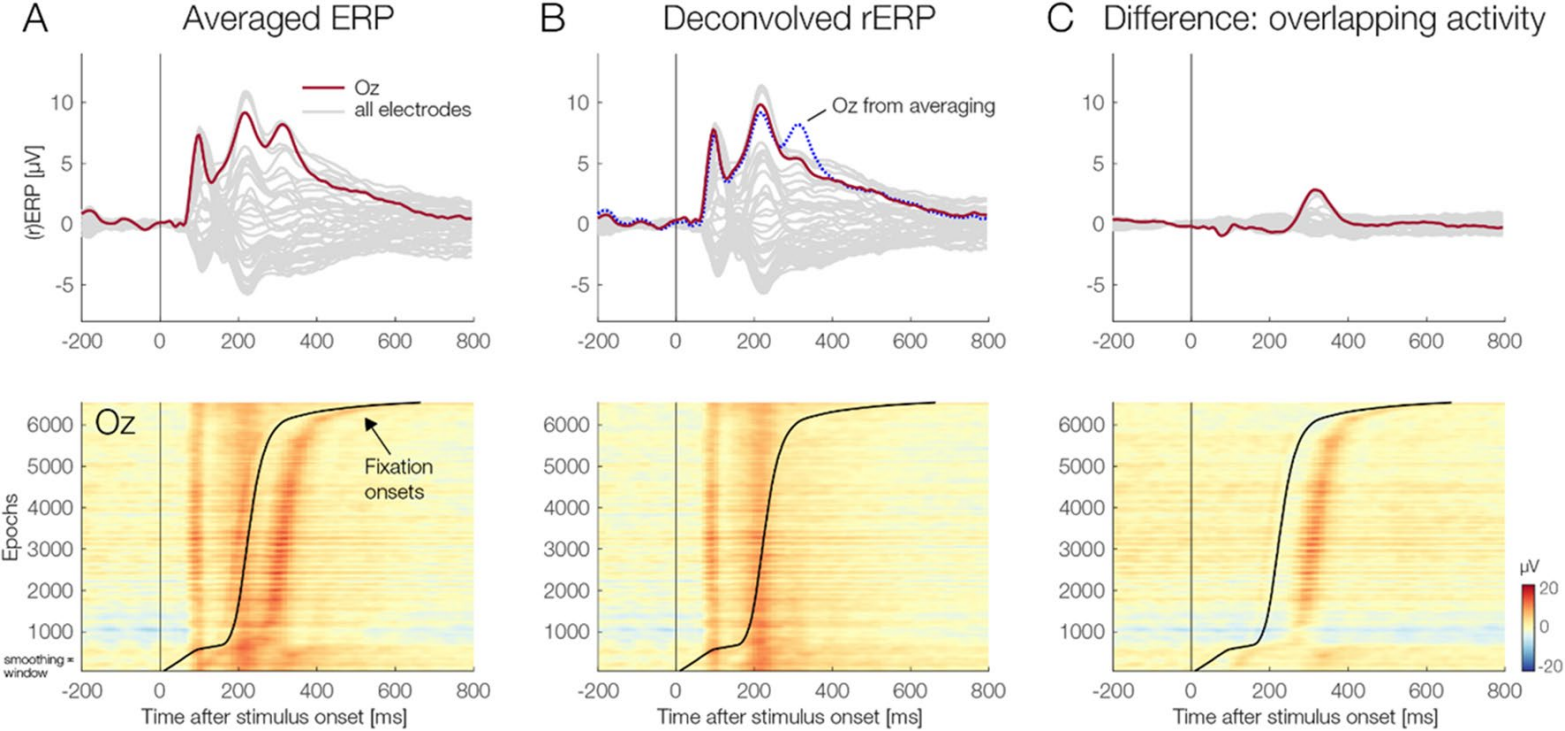
Comparison of ERPs aligned to face onset, obtained with classic averaging (Averaged ERPs) and with deconvolution (Deconvolved rERPs). **A** Averaged ERPs show a distortion at around 300 ms (upper panel). The lower panel shows the latency-sorted single-trial EEG epochs (erpimage) at electrode Oz relative to the face stimulus onset. Trials were sorted by the onset latency of the first (re)fixation on the face (black sorting line). For this visualization, the erpimages were smoothed vertically across 100 adjacent epochs after sorting. **B** Deconvolved rERPs. Note how these waveforms lack the distinct peak at around 314 ms (upper panel). For reference, the blue dotted line shows the data at Oz without overlap correction, as in panel ***A***. The erpimage after deconvolution (lower panel) suggest that the overlapping activity was successfully removed. Note that these latency-sorted trials of the deconvolved data include the model residuals, meaning that any unmodeled overlapping activity would remain visible here if the overlap correction was incomplete. **C** The difference between the results without deconvolution (panel ***A***) and with deconvolution (panel ***B***). In the upper panel, the difference waves show the “pure” distortion introduced by overlapping eye movement-related brain activity. Similarly, the lower panel shows the difference between the erpimages in panel ***A*** minus panel ***B***. (Color figure online)

Compared with the deconvolved rERPs, traditionally-averaged ERPs show a distinct peak at around 300 ms (Fig. 3A, upper panel), which was not present in the overlap-corrected data (Fig. 3B, upper panel). With an amplitude of 2.81 μV, this distortion was most pronounced at electrode Oz where it peaked at 314 ms poststimulus (Fig. 3C, upper panel). Since this occipital peak was eliminated by the overlap correction in the deconvolved rERPs, the measured distortion must originate from overlapping saccade-related potentials. This was confirmed by plotting the latency-sorted single-trial EEG epochs aligned to stimulus onset (*erpimages*). Without deconvolution (Fig. 3A, lower panel), we can clearly see how the confounding activity from the eye movement is superimposed on the stimulus-induced ERP. This overlapping positivity, which corresponds to the saccade-related visual lambda response, is successfully removed in the deconvolved EEG signals (Fig. 3B, lower panel). Finally, Fig. 3C shows only the “pure” overlapping fixation-related activity, which had previously distorted the stimulus ERPs.

These results confirm previous reports on the contamination of stimulus-locked ERP waveforms by overlapping brain responses from (micro)saccades (Dimigen & Ehinger, 2021; Dimigen et al., 2009). Because the first saccade on the face occurs at a similar latency in most trials, these potentials do not jitter out, but can create a considerable distortion of stimulus ERPs at occipital scalp sites.

#### Fixation FRPs

**Fig. 4:**
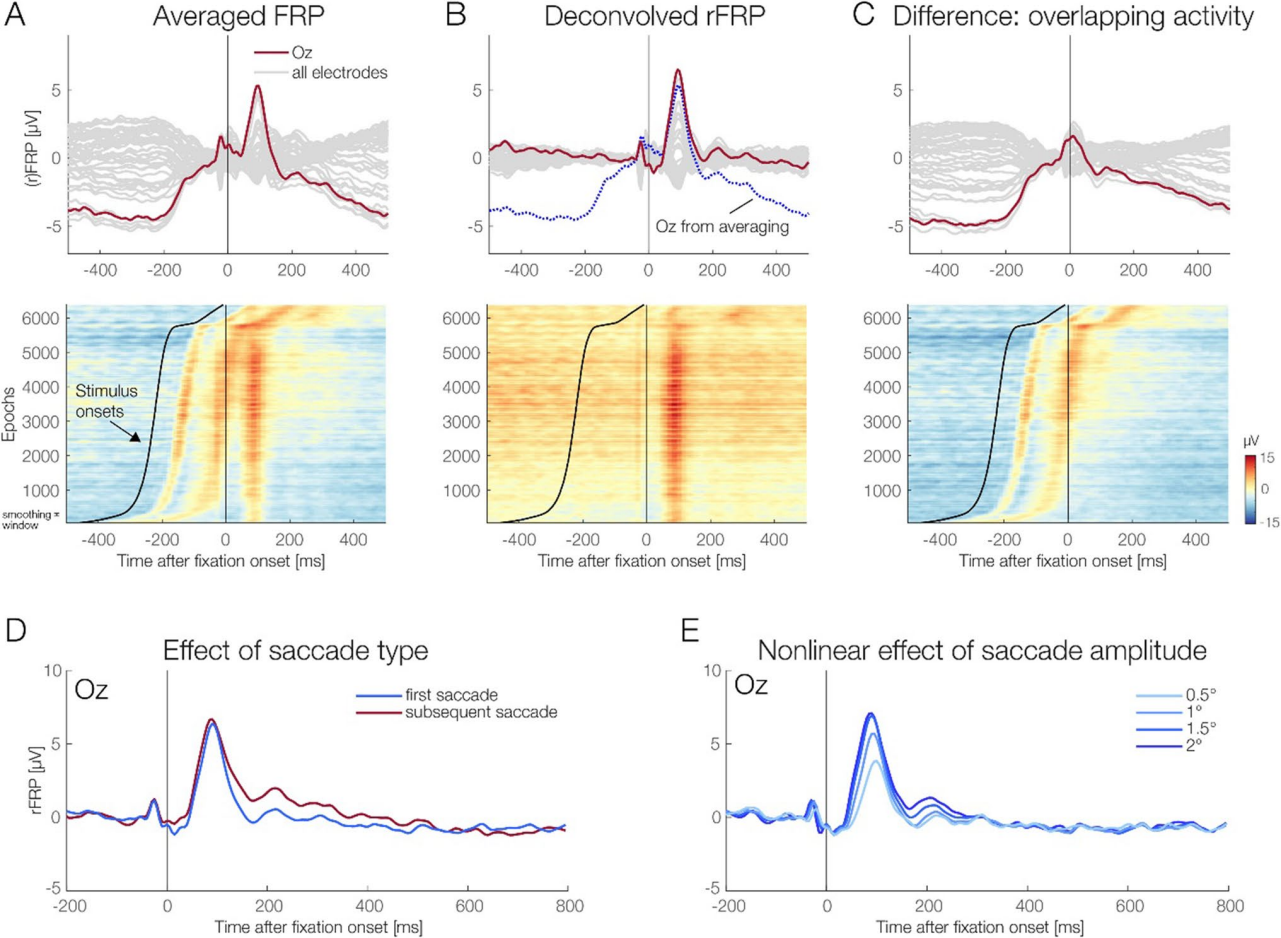
Comparison of fixation-related potentials obtained with classic averaging (FRPs) and with deconvolution (rFRPs). **A** Without overlap correction, averaged FRPs were strongly distorted, both before and after fixation onset (upper panel). The lower panel shows the underlying single-trial EEG signals at Oz relative to fixation onset (erpimage). Trials are sorted by the latency of the preceding onset of the face stimulus, as indicated by the black sorting line. Strong distortions of the FRP waveform by the preceding stimulus onset are evident. **B** Overlap-corrected rFRPs do not show these distortions anymore (upper panel). The lower panel again shows the corresponding erpimage. **C** The difference between averaged vs. deconvolved FRPs shows the “pure” distortion of the FRP waveform by the preceding stimulus onset. **D** Effects of saccade type (first vs. subsequent) on rFRPs, illustrated at electrode Oz. Following the P1 peak, rFRPs for subsequent saccades remained more positive at occipital scalp sites. **E** Effect of incoming saccade amplitude on rFRPs. Note that the P1 amplitude is almost identical for 1.5° and for 2.0° saccades, showing the strong nonlinearity of the effect. (Color figure online)

Figure 4 shows the effects of overlap correction on the brain potentials elicited by (re)fixations on the face. Compared with the overlap-corrected waveforms, the traditionally averaged FRP curves were heavily distorted, both before and after fixation onset (Fig. 4A, upper panel). This distortion resulted in a smaller lambda response (P1) at 100 ms and a strong signal drift before and after. In contrast, the deconvolved rFRPs show a clear spike potential and lambda response (Fig. 4B, upper panel). The upper panel of Fig. 4C shows the difference waves between averaging and deconvolution, again highlighting the strong distortions seen in traditionally averaged FRPs.

Deconvolution modeling also showed that the overlap-corrected rFRPs varied according to the type of the preceding saccade (first vs. subsequent) and according to saccade amplitude. Specifically, following the P1 (lambda response) peak, rFRPs elicited by the first saccade were more negative than those elicited by subsequent saccades (Fig. 4D), possibly reflecting adaptation (Auerbach-Ash et al., 2020; Gert et al., 2022). Our statistical analyses (reported in the following section) confirmed that this difference was significant, both within the EPN ROI and also in the permutation statistics (TFCE).

As expected, saccade amplitude also influenced the rFRP waveform (Fig. 4E): With increasing saccade size, the fixation-related lambda response (P1) peaked earlier with a larger peak amplitude. As observed previously (Dandekar et al., 2012; Dimigen & Ehinger, 2021; Ries et al., 2018; Yagi, 1979), this increase with saccade amplitude was nonlinear: Specifically, a 0.5° increase in saccade amplitude lead to much larger change in P1 peak amplitude within the population of small microsaccades (i.e., from 0.5° to 1.0°) than within the population of medium-sized saccades (i.e., from 1.5° vs. 2.0°).

**Fig. 5:**
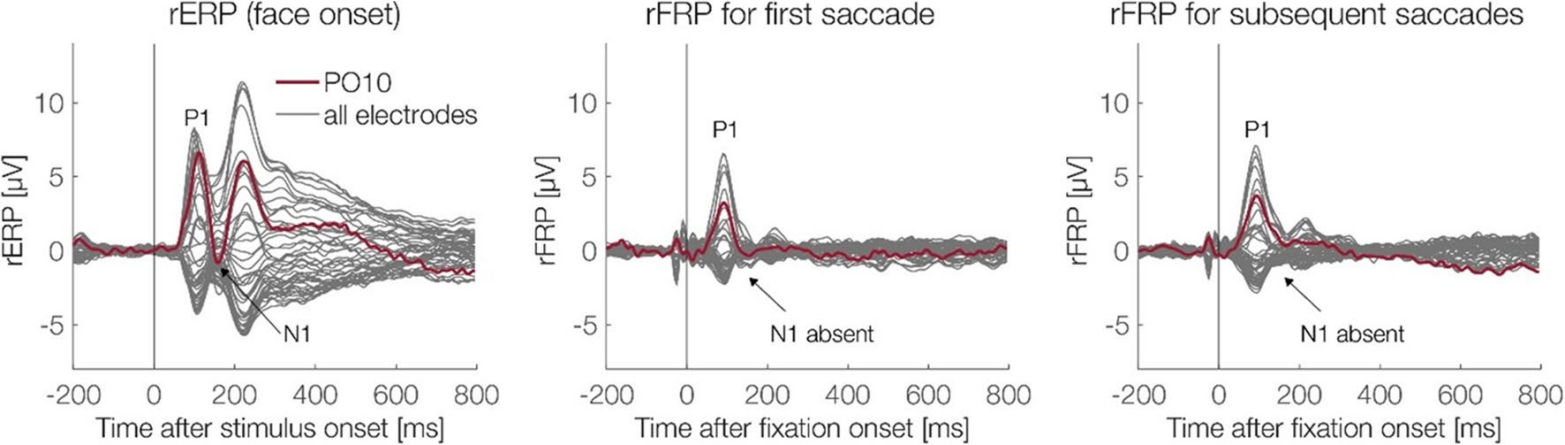
Lack of late or “endogenous” signal components in (micro)saccadic FRPs. The figure shows the grand-average overlap-corrected rERP and rFRP waveforms for the neutral condition at all EEG channels (dark-grey lines)*.* Electrode PO10, where N1/N170 effects to faces are often largest, is highlighted in red. Whereas the event-related potentials elicited by the onset of the face show a clear P1–N1 complex (left panel), the N1/N170 is strongly attenuated or absent in FRPs, both for the initial fixation (middle panel) and for subsequent fixations (right panel) on the face. (Color figure online)

Finally, Fig. 5 compares the brain potentials following stimulus onset (rERPs) with those elicited by fixations (rFRPs). One interesting difference concern the N1 component: Whereas stimulus-onset rERPs showed a clear P1–N1 complex, the N1 was strongly attenuated or even absent in rFRPs (see arrows in Fig. 5). More generally, the brain response following refixations showed a striking absence of late or “endogenous” components in the waveform.

### Emotion effects in stimulus- and fixation-related potentials

In our study, we expected emotion effects on the EPN component in both stimulus ERPs and refixation-FRPs. Therefore, as a first step, we ran a “global” ANOVA to test for emotion effects across event types—that is, including both rERPs and rFRPs. We ran this ANOVA on the average voltage in the predefined occipitotemporal region-of-interest and in the a priori defined time window (from 200 to 300 ms). For this global analysis, we also aggregated across the factor *Saccade Type* (first vs. subsequent saccades) in rFRPs. This global ANOVA revealed significant main effects of *Emotion*, *F*(2, 38) = 3.87, *p* = .03, η_G_^2^ = 0.009, and *Event Type* (stimulus onset vs. fixation onset), *F*(1, 19) = 30.36, *p* < .001, η_G_^2^ = 0.45, as well as a significant interaction effect between *Event Type* and *Emotion*, *F*(2, 38) = 5.95, *p* = .006, η_G_^2^ = 0.01. These results suggest that while there is a significant overall effect of emotion on neural responses in the EPN window, the effect also depends on whether the brain potentials are aligned to stimulus onsets or to refixations. In the next steps, we therefore analyzed the emotion effects separately within each event type.

#### Stimulus-related potentials (regression ERPs)

**Fig. 6:**
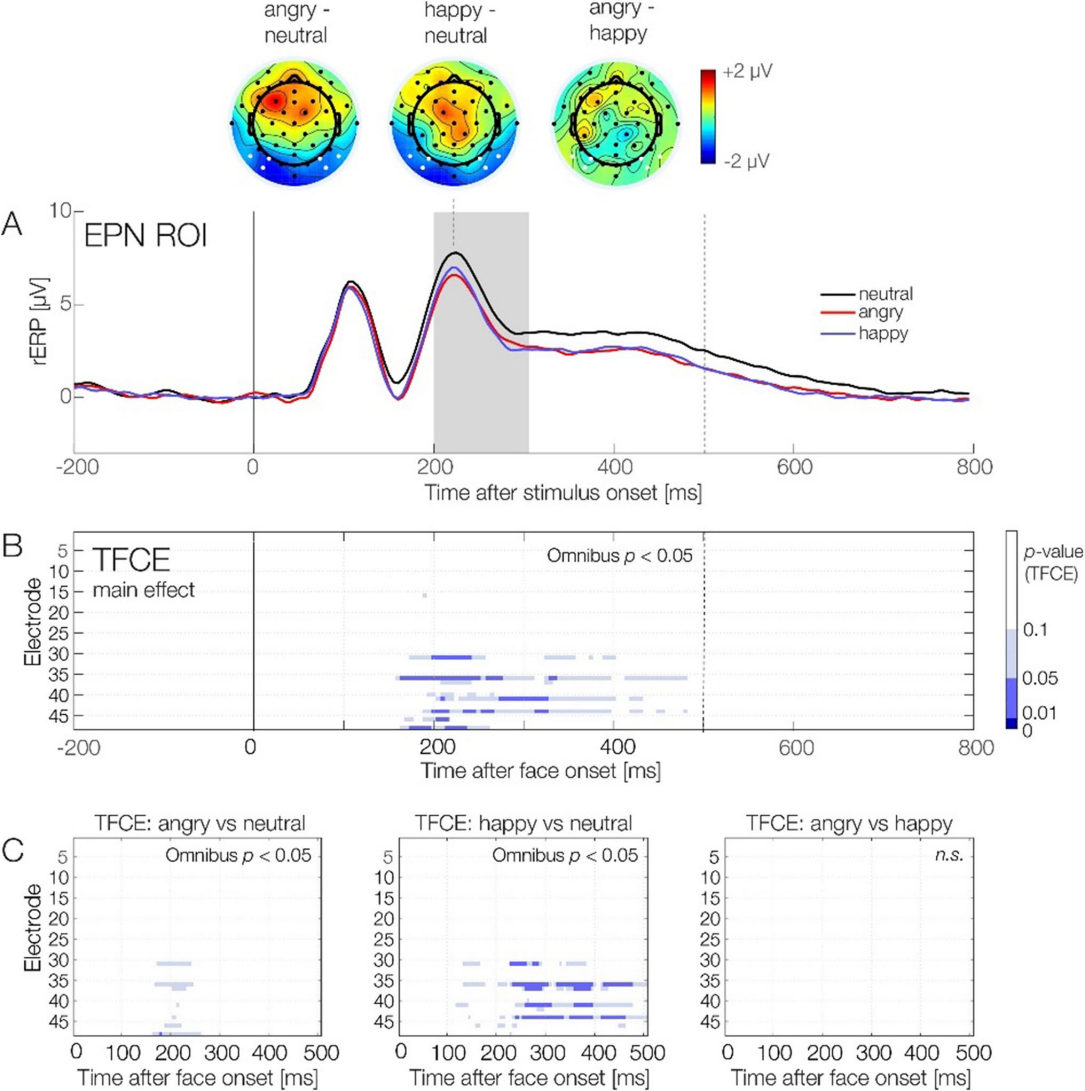
Emotion effects in overlap-corrected regression ERPs. **A** Regression ERP waveforms for the three emotion conditions, averaged over the spatiotemporal ROI for the EPN component. Difference topographies contrasting the three emotion conditions are exemplified at the latency of 230 ms after face onset; electrodes belonging to the ROI are highlighted in white. The time window to quantify EPN amplitude (200–300 ms) is highlighted in grey. **B–C** Results of the permutation test (TFCE-ANOVA), conducted on the interval from 0 until 500 ms (dotted vertical line). **B** Shows the main effect of *emotion*; the three panels in **C** visualize the results of the post hoc TFCE-based *t* tests comparing the three facial expressions (angry, happy, neutral). The tests confirm the presence of an emotion effect, which distinguishes both the happy and the angry condition from the neutral condition. The contrast between the angry and happy condition was not significant. (Color figure online)

Figure 6 depicts the rERPs elicited by the stimulus onset as a function of emotion condition. Consistent with our hypothesis, rERP amplitudes in the EPN window were more negative for angry and happy as compared with neutral faces. Difference topographies contrasting the three emotion conditions, shown at the top of Fig. 6, confirm the EPN-typical bilateral occipitotemporal negativity for the two contrasts between the emotion conditions (happy and angry) minus the neutral condition.

We statistically tested for emotion effects in rERPs in two ways: Firstly, we conducted ROI-based analyses based on our a-priori hypotheses regarding the EPN component. Secondly, we used a permutation test (TFCE-ANOVA) that allowed us to also identify possible emotion effects across all channels and time points. We used this second approach because the spatiotemporal properties of emotion effects in FRPs (Guérin-Dugué et al., 2018; Simola et al., 2013, 2015) are not yet as well-established as those in ERPs.

The classic (frequentist) ANOVA on the EPN ROI (200–300 ms) revealed a significant main effect of *Emotion*, *F*(2, 38) = 5.26, *p* = .009, η_G_^2^ = 0.03. Post hoc frequentist *t* tests revealed a significant difference between happy and neutral faces, *t*(19) = 2.82, *p* = .03, Cohen’s *d* = 0.63. Neither angry versus neutral, *t*(19) = 2.61, *p* =.051, nor angry versus happy, *t*(19) = −0.03, *p* = 1.00, differed significantly. Subsequently, we used a Bayesian ANOVA to quantify how much evidence there is for our hypothesized emotion effect in rERPs (and rFRPs, see next section). We found moderate evidence towards the alternative hypothesis that there is an emotion effect (Bayes factor *BF =* 4.88, ±0.68%). An error percentage of 0.68% suggests strong robustness of the resulting Bayes factor (van Doorn et al., 2021 recommend percentages below 20% as acceptable; our percentages are well below this threshold).

Figure 6B shows the results of the permutation test (TFCE-ANOVA) on the within-subject factor *Emotion*. Convergent with the ANOVA for EPN window, the main effect *Emotion* also reached significance in this test (peak significance: PO9 at 205 ms): *F*(2, 38) = 9.81, *p* = .03. Inspection of the TFCE-ANOVA plots (Fig. 6B) suggests that the overall effect in this test is driven by a cluster of parieto-occipital electrodes (see Fig. 6A) which show more negative amplitudes for angry and happy compared with neutral faces beginning at around 160 ms after face onset and lasting for several hundred milliseconds. Post-hoc TFCE *t* tests, visualized in Fig. 6C, confirmed a significant difference between happy and neutral (peak significance observed at PO9 at 270 ms), *t*(19) = 3.67, *p* = .03, with amplitude difference of *M* = −1.38 μV, and between angry and neutral (peak significance at M1 at 175 ms), *t*(19) = 4.65, *p* = .049, amplitude difference of *M = −*1.20 μV. Angry and happy did not differ significantly in the permutation test (*p* = .56).

Taken together, using both classic ROI-based and TFCE-based ANOVAs, we found significant emotion effects in stimulus-locked regression ERPs. Bayesian analyses suggest moderate evidence for emotion effects in rERPs. As expected, frequentist post hoc *t* tests (both based on the EPN ROI and based on TFCE) revealed significant differences between happy versus neutral faces, whereas the happy and angry conditions did not differ. When comparing angry versus neutral faces, TFCE revealed a significant difference whereas the EPN ROI analyses did not. However, the *p* value of latter test was close to our pre-defined significance threshold (*p* = .051), and the TFCE results suggest that angry and neutral differed at time points (peak: 175 ms) and electrodes (left mastoid, M1) that were slightly outside of our pre-defined spatiotemporal ROI.

#### Fixation-related potentials (regression FRPs)

**Fig. 7:**
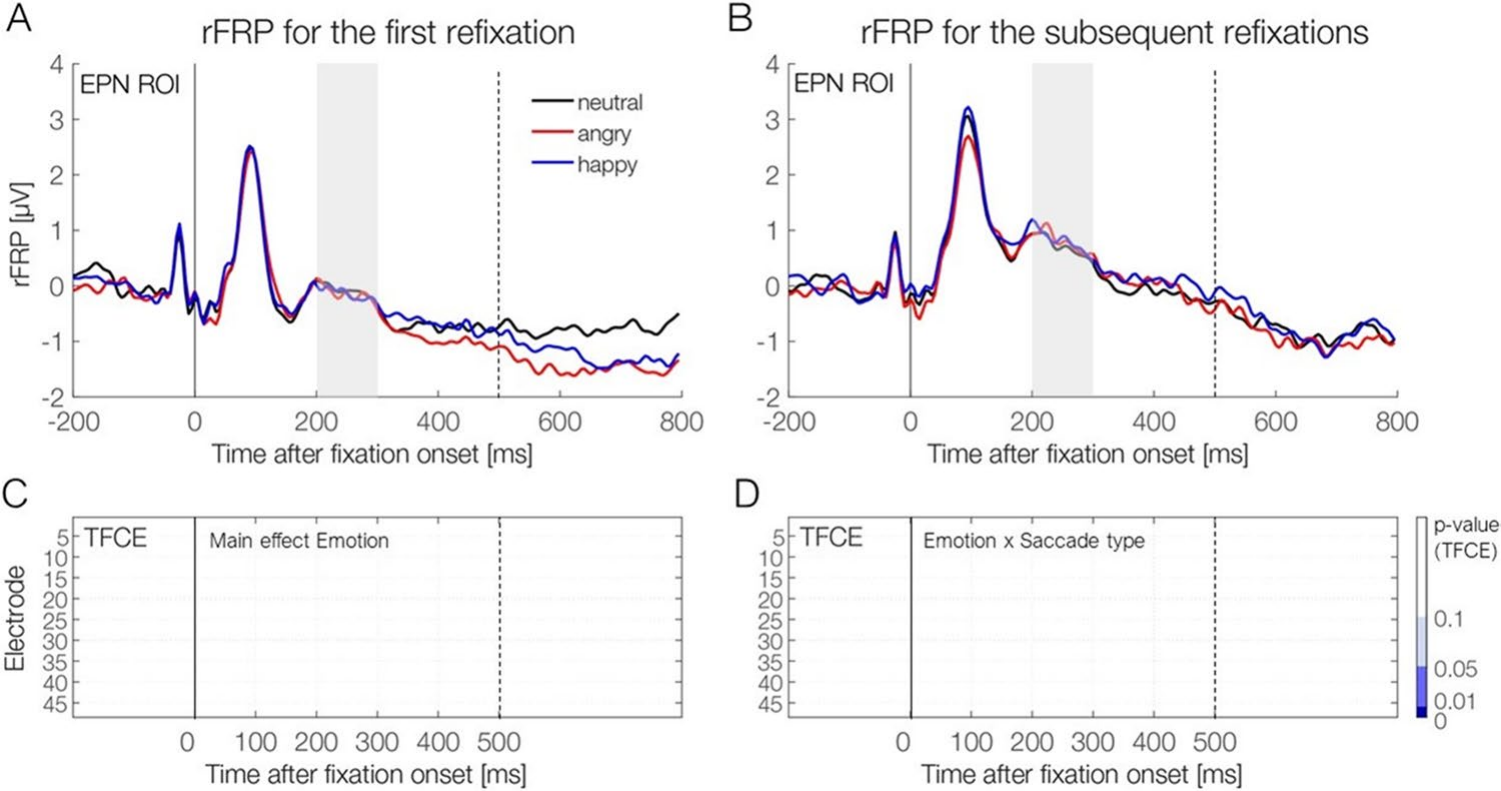
Regression-FRP waveforms for the three emotion conditions for the first refixation **(A)** and subsequent refixations **(B)** on the face. Waveforms are shown averaged across the occipitotemporal ROI for the EPN component. The EPN time window (200–300 ms) is highlighted in grey. **C–D** Results of the cluster permutation test (TFCE-ANOVA), conducted on the interval from 0–500 ms. ***C*** Shows the nonsignificant main effect of Emotion, and panel ***D*** the nonsignificant interaction Emotion × Saccade Type (first vs. subsequent saccade). These results provide no evidence for significant emotion effects on rFRPs, regardless of whether the fixation followed the first saccade or a subsequent saccade on the face. (Color figure online)

Figure 7 depicts the rFRP curves as a function of *Emotion* and *Saccade Type*. To test for emotion effects in rFRPs, we again used a ROI-based approach and a permutation approach (TFCE). The classic frequentist ANOVA analysis in the EPN ROI (occipitotemporal electrodes, 200–300 ms) revealed a significant main effect of *Saccade Type, F*(1, 19) = 27.88, *p* <.001, η_G_^2^ = 0.06. This main effect of *Saccade Type* is also illustrated in Fig. 4D and shows that the first refixation elicits more negative amplitudes at an occipital electrode (Oz) than subsequent saccades do. We found no main effect of *Emotion, F*(2, 38) = 0.02, *p* = .98. The interaction between *Saccade Type* and *Emotion* was not significant either, *F*(2, 38) = 0.26, *p* = .73. Using a Bayesian ANOVA in the same spatiotemporal ROI, we found moderate evidence against the hypothesis that there is an emotion effect in rFRPs (*BF =* 0.14, ±1.81%). Again, an error percentage of <2% suggest strong robustness of this Bayes factor.

The TFCE-ANOVA, with *Emotion* and *Saccade Type* as within-subject factors, yielded converging results. The TFCE-ANOVA on rFRPs revealed a significant main effect of *Saccade Type* (peak significance observed at electrode Iz at 160 ms; result not plotted here), *F*(1, 19) = 67.51, *p* < .05. However, there was no significant main effect of *Emotion* on rFRPs (*p* = .55), nor a significant interaction between *Emotion* and *Saccade Type* (*p* = .87). Taken together, while we find a classic emotion effect on the EPN in stimulus-evoked potentials, both ROI-based and permutation-based analyses provided no evidence that refixation-rFRPs are modulated by the emotional expression of a face.

## Discussion

The active oculomotor exploration of the environment continues at a miniaturized scale and at a somewhat slower pace also during traditional EEG experiments. Previous work indicates that potentials from (micro)saccades made during the experimental trials may not just be a hidden source of artifacts (Yuval-Greenberg et al., 2008), but also a source of useful information (Guérin-Dugué et al., 2018; Meyberg et al., 2015). In the current work, we applied linear deconvolution techniques to the EEG data of a traditional face recognition experiment to separate brain potentials elicited by the stimulus presentation from those generated by small gaze shifts within the face. We hypothesized that each of these refixations would produce a volley of visuocortical activity (Dimigen et al., 2009) and we were interested whether an established ERP effect—that of emotional valence on the EPN component—would also be reflected in the resulting fixation-related potentials (FRPs). Specifically, we hypothesized that this might be the case for the first refixation on the face, which usually occurs only around 200–250 ms after stimulus onset and therefore in the same latency range that the EPN emerges in stimulus-locked ERPs.

In our experiment, participants viewed emotional faces for 2 seconds with the task to report occasional gaze changes within the stimulus face. As expected, we found that the rather small (median: 1.23°) saccades on the face produced sizeable brain responses, with amplitudes that were at least similar to those elicited by the stimulus onset, at least for the P1 component (cf. Fig. 5). Importantly, linear deconvolution allowed us to fully disentangle the stimulus ERPs from the following FRPs and vice versa. However, although we replicated the expected EPN effect of facial emotion in the (overlap-corrected) stimulus ERPs, such an effect was not observed in the FRPs elicited by subsequent (micro)saccades on the face. In the following, we will discuss our results in more detail, relate them to existing research, and provide an outlook for future research.

### Stereotypical saccades within the face were found in nearly every trial

Although a fixation cross was shown prior to face onset, participants made small saccades within the eye region of the face in virtually every (98%) trial. Following face onset, the saccade rate exhibited the common dynamic of an initial saccadic inhibition followed by a rebound (Engbert & Kliegl, 2003; Reingold & Stampe, 2002). Saccade rate reached a minimum at around 120 ms, but then increased strongly, with the first (micro)saccade typically happening after around 200 ms (Fig. 2D).

Interestingly, the emotional expression shown by the face had only a minimal impact on these eye movements. While previous studies have shown that emotion can influence eye movements when participants are asked to categorize facial expressions (e.g., Eisenbarth & Alpers, 2011), our gaze shift monitoring task produced very limited differences in oculomotor behavior. Particularly, saccades on angry faces were slightly smaller and their vertical landing positions were distributed slightly differently within the face (Table 1), but unstandardized effects sizes were small. Overall, we found that the participant’s eye movements were highly stereotypical: Beginning at the bridge of the nose, the first saccade was usually aimed at one of the eyes, typically the left one (from the perspective of the observer). While this gaze behavior was of course adaptive for the current change detection task, we observed similar stereotypical gaze behavior also in a previous experiment that required emotion classification (see Experiment 1 in Dimigen & Ehinger, 2021). We suspect that the repetitive presentation of hundreds of highly standardized face stimuli (with no external features, shown at an identical screen location) explains this repetitive oculomotor behavior seen in EEG experiments on face recognition. Our finding that the size and direction of (micro)saccades was overall highly similar between emotion conditions should be reassuring for face researchers who are concerned about confounds from differences in gaze behavior between conditions (e.g., Vormbrock et al., 2023).

As mentioned, participants exhibited a strong preference for looking at the left eye of the stimulus face, which likely reflects a previously reported bias to prioritize information on the left side of another person’s face (e.g., Burt & Perrett, 1997; Butler et al., 2005; Gilbert & Bakan, 1973; Vinette et al., 2004). This bias also manifests itself in eye movements, where the first saccade on a face is often aimed towards the left (Butler et al., 2005; Butler & Harvey, 2006; Hsiao & Cottrell, 2008; Mertens et al., 1993). Notably, however, some of this effect may not be specific to faces, but may reflect a more general tendency to direct the first saccade on complex stimuli towards the left (e.g., for natural scenes see Nuthmann & Clark, 2023; Nuthmann & Matthias, 2014), a phenomenon which has been hypothesized to reflect a relative dominance of right-hemispheric parietal-frontal attention networks (“pseudoneglect”; Bowers & Heilman, 1980). Regardless of the exact cause of this leftward bias, we interestingly observed that it was already present for microsaccades during the prestimulus baseline interval (see Fig. 2C, left panel) suggesting that there is an anticipatory attention shift towards the preferred left side already before the face is even shown.

In summary, we found that participants executed one or more small saccades towards the task-relevant eye region of the face in almost every trial. Most eye movements occurred stereotypically and rather synchronously shortly after the start of the trial, with only marginal differences in oculomotor behavior between emotion conditions.

### Deconvolution cleanly isolates stimulus- from fixation-related potentials

Our first research aim was to use (non)linear deconvolution to separate ERPs from FRPs. To this end, we first compared traditionally-averaged ERPs with overlap-corrected regression ERPs (rERPs). Over posterior scalp sites, averaged ERPs showed a large distortion of 2.81 μV from overlapping saccades peaking at 315 ms poststimulus. The latency and amplitude of this distortion originated from the highly synchronous first saccade (at ~200 ms) which elicited a large lambda response about 90-100 ms later. A similar impact of microsaccades on the P300 amplitude has previously been demonstrated (Dimigen et al., 2009). Importantly, after overlap correction, rERPs did not show this distortion anymore. Instead, the overlapping activity was cleanly separated, as evident by the absence of residual saccade-related activity in the latency-sorted single trials after deconvolution (see Fig. 3B).

Secondly, we examined whether deconvolution can successfully isolate an rFRP waveform with a clear P1 (lambda response) and N1 component. As expected, without deconvolution, FRP waveforms were massively distorted by overlap (Coco et al., 2020; Gert et al., 2022). In contrast, deconvolved rFRPs showed a clean and rather flat baseline without any of these distortions (Fig. 4D). We conclude that (non)linear deconvolution can successfully separate saccade-locked activity from stimulus-locked activity (see also Devillez et al., 2015; Dimigen & Ehinger, 2021; Gert et al., 2022; Kristensen et al., 2017).

In addition to the *Emotion* factor, we included saccade amplitude as a nonlinear spline predictor in the model. Our results confirm a previously reported nonlinear relationship between saccade size and FRP amplitude (Dandekar et al., 2012; Dimigen & Ehinger, 2021; Dimigen et al., 2011a; Dimigen et al., 2009; Ries et al., 2018; Thickbroom et al., 1991; Yagi, 1979). As shown previously (Dimigen & Ehinger, 2021), for unknown reasons, the influence of saccade size is highly nonlinear for the lambda response but more linear for later intervals of the rFRP waveform after about 150 ms (see Fig. 4E). From a methodological viewpoint, these findings emphasize the importance of including saccade size as a nonlinear predictor in the model (Dimigen & Ehinger, 2021).

### Face onset-ERPs are enhanced by emotion, but refixations may only reflect lower-level visual processing

Our second research question was whether both stimulus and fixation-related potentials would show an EPN effect of emotion. More specifically, we wanted to examine whether FRPs are enhanced by the reflex-like allocation of additional processing resources believed to underlie the EPN effect for arousing stimuli. In potentials time-locked to face onset, we observed the expected EPN effect from 200-300 ms, with more negative voltages at occipitotemporal electrodes for angry/happy faces as compared with neutral faces. Both the scalp distribution and timing of this effect resemble the EPN previously reported in the literature (Schindler & Bublatzky, 2020). The effect was also found in the cluster permutation test. In line with previous research (Rellecke et al., 2011, 2012), we found this EPN effect despite the fact that emotion was task-irrelevant. Although we did not formally test for emotion effects on the earlier N170, more negative voltages for angry and happy facial expressions were already seen during the peak of the N170 (see Fig. 6A). It remains unclear whether this apparent effect is functionally distinct from the later EPN effect (Rellecke et al., 2013). There was no evidence that the later LPP component was modulated by emotion, which is expected since our experiment did not require an emotion classification.

The core question was now whether a similar EPN effect—or any other effect of facial emotion—would also be seen in the brain responses elicited by refixations. This did not seem to be the case. The frequentist statistical analyses of rFRPs did not show a significant EPN effect of emotion, neither in rFRPs following the first saccade on the face, nor following subsequent saccades. The cluster permutation test, while less sensitive, likewise did not provide evidence for effects at other channels or time points. In frequentist statistics, the absence of a statistically significant result does not necessarily imply the absence of an effect. Therefore, we employed Bayesian statistics, and Bayes factors specifically, which allowed us to quantify the amount of evidence in favor of the null over the alternative hypothesis (or vice versa). Our supplementary Bayesian analysis of the data found moderate evidence for the null hypothesis that there was no influence of emotion on rFRPs. In contrast, for the stimulus-locked rERPs, the Bayesian analysis provided moderate evidence in favor of the alternative hypothesis (emotion influences rERPs).

In summary, our results are consistent with the notion that once participants had been exposed to an emotional static face, they did not reprocess facial emotion when refixating it about 200 ms later. This apparently rapid processing of emotional facial expressions may seem surprising given that face recognition seems to involve more than just a single fixation (Hsiao & Cottrell, 2008). One likely interpretation of the current result is that the reflex-like allocation of more processing resources assumed to underlie the EPN effect only occurs once, in response to the initial stimulus exposure. This would be reminiscent of the rapid categorical adaptation of the face-vs.-object effect in FRPs (Auerbach-Asch et al., 2020; Gert et al., 2022) where a large N170 is only observed during the first fixation of a face, but not during an immediately following fixation of a (different) face stimulus. Similarly, the absence of emotion effects in rFRPs in the present study may also reflect a rapid neural adaptation to facial emotion in the form of repetition suppression (Kovács et al., 2006), which describes the phenomenon that neurons show a suppressed response to repeated stimuli to which they are sensitive (e.g., see Caharel et al., 2009 for effects on the face N170). In future research, it would be interesting to directly compare the effect of (micro)saccade-induced re-fixations to the effect of a repeated passive stimulation with the same face stimulus in the absence of eye movements.

An alternative possibility is that the potentials elicited by microsaccades may generally be restricted to early cortical stages of the visual pathway. This interpretation would be consistent with the observation that the rFRPs in our study show a strong lambda response (P1), but that later, “endogenous” ERP components were seemingly attenuated or absent, including the N1/N170 component (see Fig. 5). Finally, the absence of significant emotion effects on FRPs in our study may be due to our only moderate sample size of 20 participants. It is possible that a larger participant sample would be needed to uncover more subtle emotion differences in FRPs, at least in the current paradigm.^4^

Of course, whether or not microsaccadic potentials show attentional, affective or cognitive modulations may also strongly depend on the situation. In the current experiment, faces were static and facial emotion was both easy to process and task-irrelevant. In the future, it would be interesting to investigate whether FRPs become sensitive to facial emotions in contexts in which the emotion of another person’s face is more difficult to recognize (e.g., faces presented at low contrast, faces showing more ambiguous facial expressions such as contempt at a medium intensity) or in which the emotional expression is changing over time (dynamic facial expressions). In these settings, eye movements might serve the purpose to resolve uncertainty about the emotional content of a face, thereby eliciting brain responses sensitive to emotion. Along these lines, and in contrast to our findings, Guérin-Dugué et al. (2018) reported an effect of emotional facial expressions on regression FRPs during the free viewing of more complex and naturalistic face images (which also included external features such as the ears, hair, and some clothing). Specifically, these authors found significant differences between surprised and neutral faces, but only on the lambda response (P1) and on the P2 component of FRPs elicited by the first refixation. There were no effects for the other emotion contrasts, e.g., those involving happy or disgusted faces. Surprisingly, these authors also did not observe any traditional stimulus-locked emotion effects (on the P1, N170, P2–P3, or LPP components) elicited by face onset. One possibility to explain these discrepant findings might be the relative difficulty of extracting emotional cues from the faces in both studies. It is possible that the reflex-like sensory enhancements assumed to underlie the EPN only happen once during stimulus processing and that the timing of this enhancement depends on how difficult it is to decode the facial expression.

### Outlook

At a methodological level, our results show that existing techniques for EEG deconvolution modeling allow for a clean separation of stimulus-locked activity from the substantial but often unnoticed potentials generated by small gaze shifts on the stimulus. This not only makes it possible to eliminate potential confounds from stimulus-locked EEG measures, but also to extract multiple event-related brain responses from each trial of typical experiments. In the current work, we studied effects of emotion as a proxy to investigate the broader question of whether (micro)saccadic potentials contain psychologically meaningful information (Meyberg et al., 2015). Although we did not observe such an effect for the EPN component, it would be intriguing to examine similar effects in future research. For instance, it would be interesting to investigate whether other effects in higher-level vision, such as the N170 face inversion effect (Huber-Huber et al., 2019) are also limited to the initial stimulus presentation or whether they recur for subsequent refixations. In other contexts, it may be possible to replace externally flashed probes with microsaccadic potentials—for example, while probing the neural networks underlying working memory maintenance (Wolff et al., 2015). We hope that our study provides a useful framework to explore (micro)saccade-related brain activity in various contexts in the future.

## Footnotes

1 Small saccades are very brief, so it is of relatively little consequence whether the EEG signal is aligned to the beginning or to the end of the movement. For the present work, we aligned the EEG signals to saccade offsets—that is, to the beginning of the (re)fixations produced by the saccades (fixation-related potential [FRP]).

2 In the current work, we do not attempt to make a distinction between microsaccades and small exploratory saccades on the stimulus, but collectively refer to both as (micro)saccades.

3 Note that there is also evidence for topographically similar earlier effects of emotional expressions in the latency range of the N170 (Eimer & Holmes, 2007; Hinojosa et al., 2015). However, some of these effects may not reflect a true modulation of the N170, but temporal overlap with the EPN (Rellecke et al., 2013). While we focus on the EPN, we also tested for emotion effects across all latencies and channels.

4 In a Bayesian statistical framework, a smaller sample size means that the selected prior has a greater influence. Therefore, for our Bayes factors, we used Cauchy distributions, which are typically used as a weakly informative prior. Small samples may also lead to less robust results. To mitigate this, we reported the error percentages of the Bayes factors as a measure of numerical robustness. Here, we found that our error percentages are small (below 2%), indicating the robustness of our Bayesian results.
